# Dead but not gone: the interplay between the programmed cell death process and surrounding bacteria

**DOI:** 10.1128/iai.00509-24

**Published:** 2026-01-21

**Authors:** Sam Benson, Christopher J. Anderson

**Affiliations:** 1 Centre for Inflammation Research, Institute for Regeneration and Repair, University of Edinburgh, Edinburgh, United Kingdom; University of Pittsburgh, Pittsburgh, Pennsylvania, USA

**Keywords:** cell death, apoptosis, pyroptosis, necroptosis, ferroptosis, NETosis, gut microbiota, oral microbiota, efferocytosis

## Abstract

Cell death is an integral part of homeostasis, removing damaged and infected cells and replenishing healthy cells. It is a process well understood from a host perspective, with clearly delineated pathways and an expansive literature as to how it interacts with other immune and tissue mechanisms. However, the interaction between cell death and the microbial community is less well explored. There is an understanding of how bacterial pathogens are able to induce death and can have a detrimental impact on tissue resolution and repair but little on how bacteria respond to homeostatic cell death or death caused by non-bacterial stimuli. This review will cover recent advances in the understanding of host-microbe communication during cell death and will discuss how bacteria modulate/are modulated by cell death-related phenomena. The interplay between the microbiota and the fundamental processes involved in host cell death presents an exciting opportunity to discover how modulation of host mechanisms can beneficially modulate the microbiota, and therefore concurrently offer potential routes to control a number of conditions that have been linked to aberrant microbiota composition, including inflammatory bowel disease and cancer.

## INTRODUCTION

The daily homeostatic turnover of cells going through programmed cell death leaves the surrounding environment to inherit a cavalcade of molecules, triggering responses from adjacent tissues and the body’s immune system (1). This inheritance is multiplied when tissues undergo damage, be that from injury, inflammation, or infection that amplifies the extent of programmed cell death. The cell death process, at its core, involves the initiation of death, the communication to the surrounding tissue that death is occurring, an immune response to death, the containment and clearance of dying cells, and subsequent restoration of tissue homeostasis. A number of reviews have been produced of late covering how the body responds to the molecular and physiological inheritance from such damage to return to homeostasis, including how the body clears the dead cells (2, 3) and invokes repair mechanisms by the surrounding tissue (4, 5). However, a significant component of the response to cell death goes underreported—the response of the local bacterial community. This is of particular interest in epithelial tissues with a prominent bacterial population, i.e., the skin and gastrointestinal (GI) tract.

In the context of pathogenic bacterial infection, there has been a long-standing discussion over whether to view the concept of host programmed cell death as a pathogen adaptation strategy or host defense mechanism, a notion that was reviewed recently by Cooper et al. (6). While the biology is more nuanced than a binary either/or designation, the idea behind such a discussion is critical and by no means restricted to pathogenic bacteria. Given that bacteria are exceptionally adaptive to their environment and the highly conserved nature of programmed cell death across evolution (7, 8), the interplay between the two represents an avenue of research that can provide fundamental insights into both host and microbial biology.

This review will focus on how the local bacterial community affects, and is affected by, the process of host programmed cell death, including the induction of cell death, death-dependent soluble mediators, immune responses that are associated with cell death, clearance of dead/dying cells, and tissue repair process. Ultimately, we aim to shed light on the primary factors that influence how cell death can affect the inter-kingdom dynamic that often underlies tissue homeostasis and provoke a re-evaluation of focal points in the cell death cascade in the context of host-microbe interactions.

## MECHANISMS FOR EXTRACELLULAR SIGNALLING DURING CELL DEATH

To understand the context in which bacteria communicate with cell death, it is important to grasp the different forms that cell death can take and what substances are released into the immediate surroundings. Cell death can come about by programmed suicide (apoptosis, pyroptosis, necroptosis), dysregulated metabolism (i.e., ferroptosis), uncontrolled rupture (necrosis), or cell-specific death types (i.e., NETosis), all of which result in the release of different cellular contents that have their own impact on local tissue and the inflammatory response (9). These have been broadly split into two categories: anti-inflammatory/pro-resolution (apoptosis) and pro-inflammatory (pyroptosis, necroptosis, ferroptosis, NETosis). Each of these pathways results in the release of different signaling molecules to bring about a corresponding host response.

Apoptosis is a caspase-dependent “immunologically silent” form of cell death that is employed throughout the body for the homeostatic turnover of cells (10). Extrinsic apoptosis is triggered by extracellular triggers, namely the activation of death receptors, or by a lack of binding to dependence receptors on the outer membrane resulting in the activation of caspase 8. Intrinsic apoptosis is initiated by intracellular perturbations resulting in BCL-2 family-coordinated mitochondrial outer membrane permeabilization (MOMP) causing activation of caspase 9. Both intrinsic and extrinsic apoptosis converge and result in the activation of caspases 3 and 7, which instigate much of the apoptotic phenotype (11). When triggered, the apoptotic cell releases “find-me” signals that act to recruit phagocytes to the surrounding area to clear the apoptotic corpse, which has traditionally been suggested as a containment measure to prevent pro-inflammatory necrosis (12, 13). These “find-me” signals encompass proteins as well as the secretion of a number of small molecules/metabolites including lysophosphatidylcholine, sphingosine-1-phosphate, and nucleotides including ATP and UTP. The functional purpose of these apoptotic metabolites has been explored in the work of Medina et al. (14). Therein, the authors demonstrated the ability of apoptotic supernatants from thymocytes to bring about an anti-inflammatory transcriptional regulation in cultured phagocytes. Furthermore, the authors highlighted the ability of a subset of metabolites (spermidine, GMP, IMP) released via Pannexin-1 (Panx1), a caspase-activated transmembrane channel, to alleviate inflammation in murine models of both arthritis and lung transplant rejection. Panx1-derived molecules are also important for tissue repair, with the inhibition of Panx1 proving detrimental in pulmonary regeneration in mice and tail fin regeneration in zebrafish via the signaling capacity of ATP, released from apoptotic epithelial cells, to recruit and reprogram macrophages (15).

Necrotic cell death results in the rupture of the cell membrane and the passive release of immunostimulatory cellular contents (16). However, the programmed forms of necrotic cell death are more selective in the release of intracellular contents. Pyroptosis is triggered by the recognition of damage and pathogen-associated molecular patterns (danger-associated molecular patterns [DAMPs] and pathogen-associated molecular patterns [PAMPs]) and, like apoptosis, is caspase-mediated. Pyroptosis results in the formation of gasdermin-D (GSDMD) pores in the cellular membrane that destabilize membrane integrity and allow for the release of mature IL-1β and IL-18 (17). IL-1β and IL-18, which are activated from their pro forms by caspase 1, act to promote local inflammation as leukocyte mitogens and can result in systemic inflammation and fever (18). These GSDMD pores are of a size to preclude the release of internalized pathogens or organelles (~200 Å) but allow for the passive diffusion of small molecules and proteins across the permeabilized membrane (19). This has been further explored in the work of Mehrotra et al*.* who examined the secretome of pyroptotic cells using tightly controlled, genetically-inducible systems. Pyroptotic cells release oxylipins such as prostaglandin E_2_ (PGE_2_) during the death process, and PGE_2_ was shown to be particularly effective for increased wound healing and the recruitment of fibroblasts and macrophages (20). PGE_2_ is a well-studied lipid mediator, having wide-reaching impacts on the immune system (21, 22); thus, the pyroptotic progress is a biologically relevant source of potent immunostimulatory molecules beyond the classical pyroptotic cytokines (IL1β and IL18).

Necroptosis is a form of programmed necrotic cell death, regularly encountered when the apoptotic process is interrupted, in response to activation by DAMPs and PAMPs (23). Necroptosis can also be triggered in a similar mechanism to extrinsic apoptosis by ligand binding to extracellular membrane death receptors such as TNF-receptor 1 (TNFR1) (24). Necroptosis is mediated by the actions of mixed lineage kinase domain-like pseudokinase (MLKL), though the mechanism by which MLKL brings about membrane permeabilization is still not entirely clear. It is known that the RIPK1/3 pathway leads to the cleavage of MLKL to its active form, resulting in its oligomerization and transport to the cell membrane (25). It is suggested that this may either recruit Ca^2+^ and Na^+^ ion channels to the membrane or act as a pore-forming structure in and of itself (23). The group of Hendrik Ann-Jankersmit has carried out extensive studies on the secretome of γ-irradiated peripheral blood mononuclear cells, a process which invokes necroptosis, and demonstrated an increase in wound healing and angiogenesis following burn injuries (26, 27). Furthermore, the protein component within the secretome, and specifically paracrine factors, was directing increased tissue regeneration. The inhibition of individual paracrine factors did not impact this phenotype, but it was instead the cumulative effect of these factors that improved wound healing (28).

Ferroptosis is an iron-dependent mechanism of necrotic cell death (hence its etymology) characterized by a metabolic imbalance in redox activity, high reactive oxygen species (ROS) activity, elevated intracellular iron concentrations, and lipid peroxidation that brings about membrane permeabilization (29, 30). The main regulator of ferroptosis is glutathione peroxidase 4 (GPX4), which utilizes glutathione to convert lipid peroxides to lipid alcohols and helps to maintain intracellular redox balance (31). While not directly linked to innate immunity, ferroptosis initiation has been demonstrated to both modulate and be modulated by the innate immune response (32). Metabolomic analysis of the full secretome from ferroptotic cells has not currently been examined but would make for an insightful comparison with that from necroptotic, pyroptotic, apoptotic, and NETotic cells to gain a better appreciation for the scope and scale of intercellular communication capacity that results from ferroptosis.

NETosis is a form of programmed pro-inflammatory cell death that results in the expulsion of neutrophil extracellular traps (NETs), a web of decondensed DNA embedded with cytosolic and granulocyte proteins (33). Metabolomic analysis of the cellular contents and the supernatants from NETotic cells by Awasthi et al. displayed that the NETotic metabolome has increased levels of metabolites involved in the pentose phosphate pathway (PPP) and glutathione metabolism. This is related to increased rates of glutathionylation-induced inhibition of glycolytic enzymes, pushing energy production towards the PPP and therefore increasing NADPH production and ROS, which are ultimately released during NETosis (34). While first believed to be simply a method of trapping and killing extracellular pathogens, it has since been shown that NETosis occurs in sterile inflammation displaying both a beneficial role in resolving non-infectious inflammatory injury, as well as a detrimental role triggering auto-immune diseases (35).

The activation of one of these forms of cell death does not necessarily preclude the activation of another, resulting in a combination of cell death signaling. Furthermore, injuries themselves may progress through various types of cell death (i.e., acute injury-induced necrosis followed by programmed apoptosis) resulting in a rapidly changing landscape of death induced stimuli for the surrounding recipients.

This array of differentially abundant molecules released into the extracellular environment following cell death has only just begun to be explored for the capacity to promote intercellular host communication. Given the close proximity between host and microbe within mucosal tissues, it stands to reason that the death-dependent release of soluble factors, particularly metabolites, may influence the local bacterial community and suggest that particular bacterial populations may preferentially sense and respond to specific death pathways during health and disease.

## BACTERIAL MANIPULATION OF CELL DEATH PATHWAYS

**Fig 1:**
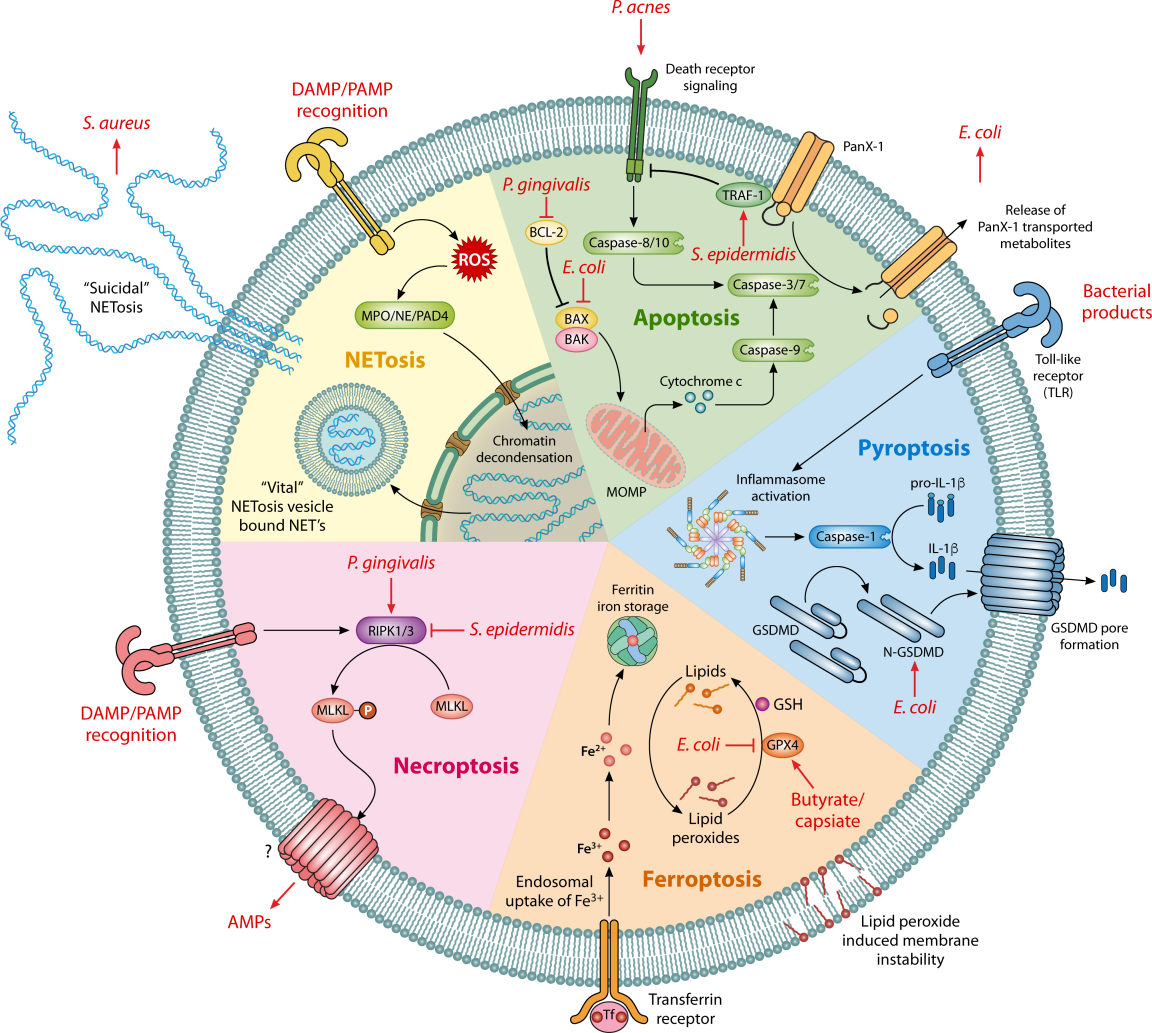
Prominent programmed cell death pathways include the major sources of extracellular metabolite release. Bacteria/metabolites that can exploit/modulate these pathways are given in red with arrows facing away, lines indicating inhibition and arrows pointing towards bacteria indicate utilization. Table 1 provides further details and references. DAMP; danger-associated molecular pattern. PAMP; pathogen-associated molecular pattern. MOMP; mitochondrial outer membrane permeabilization. AMPs; antimicrobial peptides. GSH; glutathione. Panx1; Pannexin-1 membrane channel.

Although the majority of research has focused on pathogenic organisms and the induction of particular forms of cell death, the balance of microbial species in each individual’s microbiota makes a significant difference on the outcome of cell death. For example, many commensal bacteria appear to reduce pro-inflammatory forms of cell death (Fig. 1, Table 1). In a study of allergy-induced inflammation of the nasal epithelia, inoculation with the commensal species *Staphylococcus epidermidis* reduced levels of RIPK3 and phosphorylated MLKL, thereby inhibiting necroptosis and the release of the pro-inflammatory stimulus IL-33 (36). In contrast, many pathogenic species trigger RIPK1/3-dependent necroptosis, a concept that has been reviewed recently by Yu et al. (37).

**TABLE 1:** Cell death pathways

Cell death pathway	Bacteria	Mechanism	Reference
Apoptosis	*S. epidermidis*	Inhibition – lipoteichoic acid – increased TRAF1 signaling	(38)
	*P. acnes*	Activation - TNFα	(38)
	*P. gingivalis*	Inhibition – BCL-2 upregulation	(39)
	*L. gasseri*	Inhibition – Ocludin – Tightens barrier junctions	(40)
	*E. coli*	Inhibition – OmpA – BAK, BAX, and P53 downregulation	(41)
		Utilization of apoptotic metabolites for growth	(42)
	Commensal microbiota	Activation – upregulation of Caspase 3/7	(43)
Pyroptosis	*E. coli*	Activation – GSDMD pore formation upregulation	(44)
Necroptosis	Microbiota	Secretome contains AMPs	(27)
	*S. epidermidis*	Inhibition – RIPK1/3 upregulation	(36)
	*P. gingivalis*	Activation – RIPK1/3 and MLKL upregulation	(45)
Ferroptosis	*P. anaerobius*	Inhibition – IDA – AhR receptor.	(46)
	Commensal microbiota	Inhibition – butyrate – GPX4 upregulation	(47*)*
	Commensal microbiota	Inhibition – capsiate – GPX4 upregulation	(48)
	*E. coli*	Activation – GPX4 downregulation	(49)
NETosis	*S. aureus*	Utilization of NETs for immune evasion – dAdo	(50, 51)

A similar trend is seen with ferroptosis. Commensal bacteria tend to prevent the instigation of ferroptosis, preventing a pro-inflammatory response, while pathogens tend to increase ferroptosis (52). Of course, there are exceptions, and some pathogens act to prevent the proinflammatory nature of ferroptosis (53). The aryl hydrocarbon receptor (AhR) and microbiota-derived indoles have been implicated in the impediment of ferroptosis in colorectal cancer (CRC). Trans-3-indoleacrylic acid (IDA), a tryptophan metabolite produced by the gut commensal *Peptostreptococcus anaerobius* and a ligand for AhR, initiates a signaling cascade, which results in the ferroptosis-suppressor protein-1 mediated production of coenzyme Q10 and ferroptosis inhibition (46). Additionally, patients with CRC have notably higher levels of *P. anaerobius,* which is also shown in murine models of CRC where faster rates of tumor progression are seen when mice are treated with IDA or *P. anaerobius*. The microbial metabolite capsiate likewise has an inhibitory effect on ferroptosis. Capsiate led to an increase in GPX4 levels, the inhibitory regulator of ferroptosis (48). In this case, however, decreased ferroptosis benefited intestinal ischemia/reperfusion-induced injury, which the authors correlated with their clinical findings that higher levels of capsiate have a negative correlation with rates of ischemia/reperfusion-induced injury. The microbiota-produced short-chain fatty acid (SCFA) butyrate plays a role in reducing lipid peroxidation in the intestinal epithelium. Sodium butyrate administration decreased ferroptosis in a mouse model of colitis, increasing GPX4 expression and improving barrier integrity in the colon (47). Why the microbiota may benefit from the inhibition of ferroptosis in a more general sense is open to interpretation. Host and microbe exist in a delicate balance with iron availability an essential requisite for both organisms’ viability with very few members of the microbiota being iron-independent (i.e., *Lactobacillus*) (54). Maintaining a stable status quo wherein the efflux and influx of iron between the host and microbial metabolic environment is therefore beneficial for the commensal population. As stated, pathogens are much more likely to instigate ferroptosis than commensals (53). For example, some disease-associated *E. coli* isolates decreased GPX4 expression in the terminal colon of Crohn’s disease patients (49). The rapid efflux of iron and ROS by ferroptotic cells may prove beneficial for many extracellular pathogenic species, with iron being used to support basic biochemistry and ROS being used as an electron source enabling anaerobic respiration (55). This may correlate with the effect of iron supplementation seen in infants wherein there is a significant increase in the proportion of Enterobacteriaceae and subsequent inflammation in the gut, suggesting that this family of bacteria is very capable of taking advantage of increased iron levels (56). One could suggest that inducing ferroptosis could be a method by which to increase the bacterial share of available iron.

The heterogeneity within local bacterial populations means that different modes of cell death manipulation can be occurring within the same tissue. The study of ultraviolet B (UVB) irradiation-induced DNA damage by Wang et al. provided an excellent example of how different bacterial populations promoted or inhibited cell death in epithelial tissues (38). When treated with the supernatants from either *Staphylococcus epidermidis* or *Propionibacterium acnes*, human epidermal melanocytes that had been UVB damaged were more likely to enter apoptosis following exposure to *P. acnes* supernatants. This modulation was mediated by lipoteichoic acid in the case of *S. epidermidis*, which brings about higher expression of TNF receptor activating factor 1 (TRAF1), triggering anti-apoptotic pathways. *P. acnes* supernatants instead increased expression of TNF and increased levels of apoptosis. As DNA damage is one of the highest risk factors for the development of melanoma, this suggests how integral the balance between host and the commensal bacteria can be for long-term tissue health.

A similar balance can be observed within the oral epithelium. White et al. evaluated the impact of a panel of commensal and pathogenic oral bacteria on oral epithelial cell death and concluded that commensal bacteria actively increased levels of apoptosis (43), leading to the hypothesis that commensal bacteria reduce inflammation and maintain the structural integrity of periodontal tissues. Pathogenic bacteria, such as *Porphyromonas gingivalis*, act in the opposing manner and have been shown to both trigger necroptosis by activating RIPK1/3 and MLKL (45) and inhibit apoptosis by upregulating BCL-2 (39), resulting in a pro-inflammatory immune response and an increase in the degradation of periodontal tissue.

To view the induction of cell death in a positive or negative light (for host or microbe) is highly dependent on the disease state of the host. Case in point is the effect of two different bacterial strains, both of which inhibit apoptosis in IBD and CRC systems. Di Lucca et al. examined the effect of *Lactobacillus gasseri* on epithelial barrier integrity and cellular proliferation in the context of IBD (40). Molecules secreted by a strain of *L. gasseri,* SF1183, prevented colitis *in vivo* and brought about occludin rearrangement within cell monolayers, tightened barrier junctions, and reduced apoptosis. In this context, this is seen as a beneficial outcome for the host, in that the epithelial lining is more secure, and inflammation is likely reduced. On the other hand, Mirzarazi et al. examined why the presence of particular strains of *E. coli* (B2 phylogenetic grouping) is correlated with higher rates of colorectal cancer. They noted that the outer membrane protein A (OmpA) was linked with decreasing the levels of the pro-apoptotic proteins BAK, BAX, and p53, leading to reduced levels of apoptosis and increased proliferation (41). These findings frame the microbial impact on apoptosis in relation to the increased rates of tumorigenesis and poorer outcomes for CRC patients. As well as highlighting the positive and negative aspects of microbial inhibition of apoptosis, these works demonstrate the impact of strain as well as species-specific differences, further complicating the definition of a healthy/unhealthy microbiota.

A specific disease background does not necessarily intimate that there is a single cell death pathway involved in its progression. Following injury to the gut, there is a well-known phenomenon referred to as the Enterobacteriaceae bloom, an expansion of the Enterobacteriaceae population within the microbiota and a decrease in microbial diversity (57). This is, at least in part, mediated by apoptotic release of metabolites following injury (42). However, it has been noted in a dextran sodium sulfate (DSS) model of colitis that following injury and the Enterobacteriaceae bloom, *E. coli* causes an increased activation of gasdermin D pore formation in intestinal epithelial cells, indicating increased pyroptosis (44). Both GSDMD knockout mice and gasdermin D inhibition in wild-type mice alleviate many of the symptoms of the DSS model of colitis, suggesting that pyroptotic cell death may contribute to colitis pathology. These findings suggest that although the initial response to injury may be one form of cell death (apoptosis), the resulting damage can be modified or amplified by microbial activation of a secondary cell death pathway (pyroptosis).

## MICROBIOTA MANIPULATION OF CELL CLEARANCE

**Fig 2:**
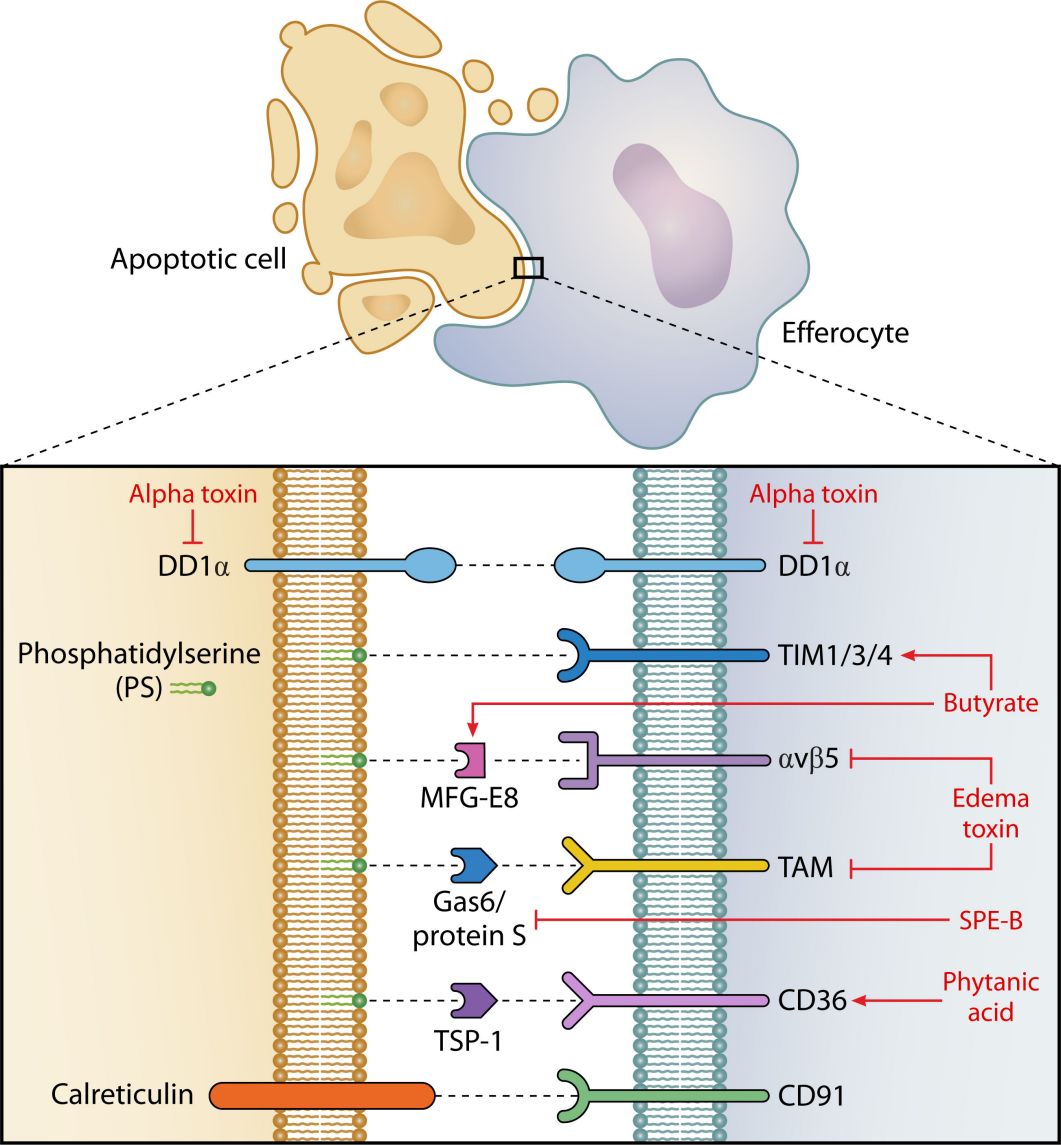
Microbial manipulation of efferocytosis. Major efferocytic receptors and their substrates in black. Bacteria/metabolites that can exploit/modulate these pathways are given in red with arrows indicating mechanism upregulation and or block lines indicating inhibition.

Following cell death, the homeostatic response to apoptosis is the efferocytosis of the apoptotic corpse to prevent the transition of the corpse into a pro-inflammatory necrotic body. This is instigated by the release of “find-me” signals by the apoptotic body via channels, such as Panx1 (58), in order to attract professional phagocytes and to alert non-professional phagocytes, such as neighboring epithelial cells, to the fate of the dying cell (59). Following this, the dying cell expresses “eat-me” signals on the extracellular membrane such as phosphatidylserine (PS), calreticulin, and DD1α which interact with phagocytic receptors on efferocytes (Fig. 2). Phagocytes express a number of corresponding receptors to identify apoptotic cells including Tim1/3/4 (recognizes PS), αVβ5 (recognizes PS via MFG-E8), TAM (recognizes PS via Gas-6 and protein S), CD36 (recognizes PS via TSP-1), CD91 (recognizes calreticulin), and DD1α (recognizes DD1α on apoptotic cells) (5). This results in the activation of the Rac1 pathway and initiates cytoskeleton restructuring enabling engulfment (60).

Metabolites from the commensal microbiota play an important part in efferocytosis (Fig. 2, Table 2). Saavedra et al. examined the impact of antibiotic usage on homeostatic efferocytosis by large peritoneal macrophages (LPMs) (61). Antibiotic usage almost completely depleted LPM efferocytosis within the peritoneal cavity but could be restored by fecal microbiome transplantation (FMT). Butyrate, a microbiota-derived SCFA, contributed towards increased levels of efferocytosis and was suggested to mediate efferocytosis via the overexpression of the efferocytosis receptor Tim4 (61). Butyrate has also been shown to increase levels of MGF-E8, another efferocytic protein, that helps bind αvβ5 to PS (62). Microbiota-derived butyrate has a long history of being implicated in changes in macrophage function and polarization, generally instigating an anti-inflammatory pro-resolution phenotype accompanied by increased efferocytosis (63). Butyrate also has a beneficial impact on intestinal mucous production, thus improving barrier integrity, and can activate macrophages to increase the levels of muc-3-expressing goblet cells (64).

**TABLE 2:** Efferocytosis

Effect	Bacteria	Mechanism	Reference
Stimulatory	Microbiota	Butyrate – increased expression of TIM4 and MFG-E8	(61, 62)
	Microbiota	Phytanic acid – increased expression of CD36	(65)
Inhibitory	*B. anthracis*	Edema toxin – decreased expression of αvβ5, TIM3, and TAM	(66, 67)
	*S. aureus*	Alpha toxin – decreased expression of DD1α	(68)
	*S. pyrogenes*	SPE-B – cleavage of protein S	(69)
	*P. aeruginosa*	Alginate sheaf / LPS	(70)

The impact of the microbiota on cell clearance can be affected by seemingly unrelated host biology. In a study of sex-based efferocytosis during lupus, male mice had higher levels of efferocytosis than females (65). The differential microbiota between the sexes was shown to be a major contributor towards this and the microbiota-derived metabolite phytanic acid produced by the male mouse microbiota specifically increased peroxisome proliferator-activated receptor gamma (PPARγ) signaling, leading to an upregulation of efferocytic receptors on macrophages. The differences in phytanic acid levels, and the differential species composition within male and female microbiota, were directly linked to the androgen differences between male and female mice, with castrated males showing a microbiota more similar to females (65). Additionally, the microbiota similarly impacts sex-driven changes in macrophage survival and differentiation (71), though it remains to be tested if this is related to efferocytic capacity.

Of course, efferocytosis is not always beneficial to members of the microbial community. A number of pathogenic bacteria express virulence factors to prevent efferocytosis and the clearance of dead cells. *Bacillus anthracis* deploys edema toxin, the combination of a protective antigen and edema factor (EF) (an adenylate cyclase), to impair macrophage efferocytosis. Edema toxin increases intracellular levels of cyclic-adenosine monophosphate (cAMP), a known inhibitor of efferocytosis, leading to decreased MerTK (a member of the TAM receptor family) and αVβ5 signaling resulting in the downregulation of Rac1 (66). *B. anthracis* peptidoglycan has also been implicated in reducing macrophage efferocytosis by similar mechanisms, reducing expression of MerTK and αVβ5 alongside a number of other pro-efferocytotic signaling receptors, such as TIM3 (67). Cohen et al*.* observed that alpha toxin released by *S. aureus* is able to inhibit the uptake of apoptotic neutrophils by alveolar macrophages (68). Alpha toxin disrupts the distribution of DD1α, a protein expressed on both dead cells and efferocytes that cross-links to initiate efferocytosis, thereby reducing efferocytosis efficiency. Alpha toxin also increases the levels of cleaved CCN1, a protein that binds to PS, indicating alpha toxin can inhibit efferocytosis using multiple pathways (68). Streptococcal pyrogenic exotoxin B (SPE B), a virulence factor released by *Streptococcus pyogenes,* likewise acts to prevent efferocytosis. SPE B acts by cleaving protein S, reducing TAM recognition efficacy, and therefore impairing clearance (69). It is not only via virulence factors that pathogens can impair the efficacy of efferocytosis. *Pseudomonas aeruginosa* produces a mucous alginate sheaf during infection to evade the immune response, and said sheaf is able to inhibit the efferocytic ability of alveolar macrophages to clear apoptotic cells (70).

## THE BACTERIAL RESPONSE TO CELL DEATH CARGO

The response of the local bacterial community, or exogenous sources of pathogens, to cell death cargo (metabolites and small molecules produced and released during cell death) has not been studied in great detail. As mentioned, cell death releases an abundance of nutrients that play critical roles in host intercellular communication, but the effect that these compounds have on bacterial populations is only just beginning to be unraveled in any detail.

Recently, the apoptotic process was shown to promote the growth of multiple members of the Enterobacteriaceae family of organisms (42). Members of the Enterobacteriaceae family, including both pathogenic and commensal organisms, sense and utilize the apoptotic-dependent metabolites that are secreted during the death process, termed death-induced nutrient release (DINNR). The apoptotic process within intestinal epithelial cells is both necessary and sufficient to promote Enterobacteriaceae growth in the contexts of infection, excessive TNF-driven inflammation, and chemotherapy-induced damage (42). Damage to the GI tract during chemotherapy has long been known to bring about dysbiosis within the commensal microbiota, particularly in the form of the Enterobacteriaceae bloom (72). This chemotherapy-driven Enterobacteriaceae bloom is, at least in part, an apoptosis-dependent effect, as caspase 3/7 deficient mice have significantly reduced Enterobacteriaceae blooms (73). Although the DINNR process itself is conserved across multiple triggers of apoptosis, the specific metabolites being released and the corresponding bacterial response slightly differ across host cell type, apoptotic trigger, and Enterobacteriaceae family member (42, 73). This heterogeneity in response is also observed during host cell-cell communication events (74), suggesting a nuanced bit of underlying biology that merits further exploration. Indeed, many of the apoptosis-dependent metabolites that are potent host cell signaling molecules are also potent promoters of bacterial growth. Metabolites released via the Panx1 channel, a caspase-dependent membrane channel, are sufficient and necessary to promote DINNR (42) and transcriptionally rewire recipient bacteria (73). Panx1 metabolites had previously been identified as being responsible for inducing immune cell recruitment to the site of damage (14), suggesting the Enterobacteriaceae are able to hijack this critical programmed host response.

The secretome of dying cells can also have an inhibitory phenotype on bacterial growth in some contexts. A combination of apoptosis and necroptosis induction in mononuclear cells can increase the proportion of antimicrobial peptides (AMP) released by the cell, such as cathelicidin and calprotectin, enabling the clearance of microbes associated with diabetic foot ulcers (27). While these AMPs were tested against pathogenic species within the wound site, one could surmise that they may have a broadly conserved impact on bacterial growth and survival.

Detailed metabolomic analyses exist for the secretomes of many forms of cell death, namely apoptosis (14), pyroptosis (20), necroptosis (26), and NETosis (34). However, the analysis of how respective metabolites impact the surrounding bacterial communities is a relatively unmined source of information. This new avenue of research can help identify critical nodes of microbial adaptation and host cell communication that may depend on the type of programmed cell death, identity of the dying “donor” cell (epithelial, immune, etc.), identity of the “recipient” cell (host, bacterial, etc.), surrounding tissue environment (intestinal, respiratory, etc.), and disease state of the host.

## BACTERIAL EXPLOITATION OF IMMUNOMETABOLISM

The microbiota can not only utilize nutrients released by dying cells but also those released by immune cells following death-dependent inflammation. Two major byproducts of the inflammatory response to lytic forms of cell death are ROS and reactive nitrogen species (RNS). These reactive species are intended as signaling molecules to modulate the immune response, as a mechanism to kill pathogens, and a means to process phagocytosed bodies. Winter et al. identified the ability of Enterobacteriaceae to utilize RNS as an electron acceptor to fuel the Enterobacteriaceae bloom. This enabled anaerobic respiration, in a molybdenum co-factor dependent manner, providing an advantageous growth mechanism in an environment dominated by obligate fermentative anaerobes (55). This has been further elucidated to show that the Enterobacteriaceae were also able to convert H_2_O_2_, using a cytochrome c peroxidase, into O_2_, which could then be utilized for aerobic respiration (75). Butyrate from the commensal microbiota has been implicated in preventing the production of RNS by epithelial cells by activating PPARγ receptors and reducing the levels of *Nos2*. Therefore, after antibiotic ablation of butyrate-producing species, *Nos2* levels rise, nitrate production increases, and the population of Enterobacteriaceae expands (76). Interestingly, apoptosis-dependent metabolites are necessary and sufficient to transcriptionally rewire aerobic and anaerobic respiration pathways in *E. coli*, even in the presence of high oxygen levels, suggesting that the bacterial sensing and utilization of different electron acceptors are linked to the host death process (73).

Inflammatory byproducts can be used as a defense mechanism, as well as a growth substrate. A number of bacteria synthesize deoxyadenosine (dAdo) from deoxyadenosine monophosphate (dAMP), including *Streptococcus suis* (77), *B. anthracis* (50), and *S. aureus (*51), to bring about the caspase-3 activated cell death of immune cells. In *S. aureus*, the combination of staphylococcal nuclease and adenosine synthase A produces dAdo, which is then taken up and converted to deoxyadenosine triphosphate (dATP) within host macrophages. The elevated levels of dATP then trigger the caspase-3-mediated apoptotic pathway (51). The work of Olaf Schneewind has exposed the particularly ingenious method by which *S. aureus* can convert the DNA within NETs produced by neutrophils during NETosis, intended to immobilize the microbe itself, to produce dAdo, both releasing the microbe from its imprisonment and eliminating those immune cells that are in pursuit (78).

While we are not covering the direct cell killing brought about by microbes, *Aggregatibacter actinomycetemcomitans* is an intriguing case of bacterial exploitation of immune cells in the context of cell death to bring about tissue damage. While the main virulence factor leukotoxin has no notable effect on gingival epithelial or fibroblast cells, leukotoxin can bring about the death of neutrophils. *A. actinomycetemcomitans* therefore actively promotes the death of neutrophils summoned to the site of infection to bring about the release of cytosolic neutrophil elastase. Neutrophil elastase then causes the detachment and cell death of the gingival cells (79). Gingival cells provide the first line of defense against pathogens, and so their destruction by the host immune system (neutrophils) allows for further invasion of periodontal bacteria.

## BACTERIAL IMPACT ON TISSUE REPAIR AND RESOLUTION

In abetting and hindering the immune response to cell death, bacterial communities also hold an important position in the wound healing process. This can be by increasing epithelial cell regeneration, stem cell proliferation, macrophage recruitment, or hampering pathogen colonization (80). On the other hand, tissue-resident bacteria can also increase inflammation, bring about biofilm formation, and invade tissues causing systemic infections (81).

The impact of the microbiota on tissue resolution is dictated by the presence of specific microbes that expand during tissue injury. Apoptosis-dependent expansion of endogenous intestinal Enterobacteriaceae significantly delays host recovery from chemotherapy-induced damage (73). Targeted antibiotic therapy, naturally Enterobacteriaceae-negative mice, and defined gnotobiotic systems demonstrated the link between Enterobacteriaceae abundance and intestinal mucositis following chemotherapy (73), which is also observed during chemically induced colitis using DSS (82). Despite the clear detrimental effects of *E. coli* within the context of a more complex/defined microbial community, germ-free mice monocolonized with *E. coli* (strain K-12) showed minimal clinical symptoms of colitis, whilst the germ-free controls all succumbed to DSS-driven disease (83). The authors determined that the purines produced in an *E. coli* monocolonized colon were primarily responsible for this lessening of disease severity, as colonic epithelial cells salvaged bacterial purines to increase host ATP production. Additionally, the introduction of exogenous hypoxanthine could recapitulate the benefit of a complete microbiota in a germ-free setting (68). In contrast, there is an increase in purine metabolites released by apoptotic cells following chemotherapeutic injury, and a corresponding reduction in bacterial *de novo* purine biosynthesis during peak dysbiosis (73). Together, these findings suggest that purines are potent signals for host epithelial cells, and the release of purines during the apoptotic process, which might be attempting to resolve tissue injury, can be intercepted by resident Enterobacteriaceae to delay tissue recovery.

The group of Luis Garza has looked extensively at the effects of the skin microbiota on wound repair, which is reviewed elsewhere (84). During wound healing in the skin, the microbiota, instead of producing metabolites, starves the area of oxygen and induces a hypoxic state that promotes hypoxia-inducible factor 1α (HIF-1α) signaling around the wound site (85). This promotes the production of glutamine in keratinocytes, promoting IL-1β production and improved hair follicle regeneration in an *in vivo* model of skin injury (86). The above works also noted how germ-free mice displayed a slower repair response to skin injury than those with a healthy skin microbiota. Uberoi et al. examined an alternative reparative pathway when they observed that keratinocytes in germ-free mice have much lower levels of AhR (87). In the same study, it was also noted that AhR knockout mice displayed low levels of tissue repair. The introduction of an AhR agonist to germ-free mice enacted an improved rate of repair that could be recapitulated upon the introduction of a human skin microbial consortium in an AhR-dependent manner. As mentioned previously, many members of the commensal bacterial population produce tryptophan metabolites and indoles that are able to activate AhR, playing roles in both cell death determination and modulating immune pathways alongside tissue repair (46, 88), emphasizing the impact of microbial metabolism in all stages of cell death and repair.

Under some conditions, it is the direct contact between the bacteria and the wound site that mediates repair, rather than the interkingdom metabolic dynamics. *Akkermansia muciniphila* is a commensal microbe that lives within the mucus layer of the gut, and it has been shown that the fecal microbiota of IBD patients have very low levels of this species (89). Wade et al. discovered that a membrane-bound protein, Amuc_1100, increased rates of wound repair and tissue resolution following DSS-induced injury (90). Mechanistically, *A. muciniphila* upregulated the transcription factor cAMP-responsive element-binding protein H, which mitigates the clinical symptoms of DSS-induced colitis. The authors elegantly showed the benefit of Amuc_1100 by inducing its production in porcine and human intestinal epithelial cells and demonstrating its ability to mitigate DSS damage in the absence of the microbe itself. Repair mechanisms are also not necessarily mediated directly by interaction with the wounded epithelial tissue itself but can be mediated by the microbiota triggering the recruitment of immune cells to the site of injury. In skin wounds, the migration of commensal bacteria into the dermis is recognized by neutrophils, which in turn recruit dendritic cells in a CXCL10-dependent manner (91). Dendritic cells are then able to promote type I interferon-mediated fibroblast repair, leading to an enhanced rate of wound closing.

## CONCLUSION

At first glance, it may be tempting to speculate that the general response of the microbiota to cell death-related mechanisms is to try and return the host to a homeostatic state. By promoting apoptosis as a form of anti-inflammatory cell death and reducing pro-inflammatory cell death mechanisms (38, 43, 47), maintaining constant levels of cell clearance via efferocytosis (61, 65), or by working to repair damaged tissues (85, 86), all of which are beneficial to the host. However, the overall outcome of cell death is complicated by the bacterial species present at the initiation of injury, strain-specific differences within said species, the type of injury, and any secondary co-morbidities that already exist.

The population dynamics within the microbiota is of obvious importance, but relatively less research is dedicated to how fundamental host cell processes and their byproducts can modulate the microbiota. This review has attempted to summarize what we know about the bacterial interaction with host cell death, a foundational mechanism occurring in the same space as commensal bacteria from birth until death. However, even in this situation, there are scant articles on the direct effects of the byproducts of cell death, clearance, and repair on commensal species. Cell death is able to fundamentally change the population of the microbiota, leading to secondary disease states and impaired tissue repair (42, 73). By unpicking these interkingdom dynamics, it is possible that this chain of events can be intercepted at an earlier point whereby the transition from a healthy microbiota can be prevented, i.e., removing the nutrient source for dysbiotic growth following cell death. What other processes could therefore similarly be having secondary impacts on overall health via the manipulation of the microbiota? There have been numerous reviews on how microbiota-derived metabolites can affect everything from kidney (92) and neurodegenerative diseases (93) to depression (94) and cancer (95). But, are there differences in the host metabolic processes that are driving the changes in the microbiota and modulating the metabolites released by commensal species causing disease? There is a forming consensus that the research community should be focusing more on the host’s responsibility in altering microbiota populations (96). This identifies that aberrant changes in host biochemistry and metabolism are a prime target for the development of new therapies for a range of microbiota-influenced diseases. It is therefore highly appealing to re-consider fundamental host processes, including the various stages of cell death listed here, for their potential to shape the microbial community.
